# Doctor, are you healthy? A cross-sectional investigation of oncologist burnout, depression, and anxiety and an investigation of their associated factors

**DOI:** 10.1186/s12885-018-4964-7

**Published:** 2018-10-26

**Authors:** Carlos Eduardo Paiva, Beatriz Parreira Martins, Bianca Sakamoto Ribeiro Paiva

**Affiliations:** 1 Department of Clinical Oncology, Barretos Cancer Hospital, Barretos, São Paulo Brazil; 2 Health-Related Quality of Life Research Group (GPQual), Learning and Research Institute, Barretos Cancer Hospital, Barretos, São Paulo Brazil; 3 Barretos School of Health Sciences, Dr. Paulo Prata – FACISB, Barretos, São Paulo Brazil; 4 Departamento de Oncologia Clínica, Divisão de Mama e Ginecologia, Rua Antenor Duarte Vilella, 1331, Bairro Dr Paulo Prata, Barretos, SP CEP: 14784-400 Brazil

**Keywords:** Cancer, Depression, Anxiety, epidemiology, quality of life

## Abstract

**Purpose:**

Doctors who work at cancer hospitals are at high risk of developing emotional distress. This study evaluated the prevalence of burnout, anxiety, and depression in a sample of oncologists of various specialties and sought to identify how much of this distress is explained by specific pre-established characteristics.

**Methods:**

This cross-sectional study used online surveys. Burnout was measured using the Maslach Burnout Inventory (MBI), and anxiety and depression were measured using the Hospital Anxiety and Depression Scale (HADS). The variables associated with *p*-values < 0.10 in the univariate analyses were included in blocks of hierarchical binary logistic regression models to identify the predictors of burnout, depression, and anxiety.

**Results:**

Of the 227 physicians (response rate = 70.5%), 132 (58.1%) were identified as having burnout (high emotional exhaustion [EE] and depersonalization [DP]); furthermore, 28 (12.3%) had depression (HADS-D ≥ 11), and 44 (19.4%) had anxiety (HADS-A ≥ 11). The block of perceptions related to the workplace explained 22.4%, 7.7%, and 10.6% of the variances of burnout, depression, and anxiety, respectively. On the other hand, the outside-of-work characteristics block explained only 3.1%, 13.4%, and 3.4% of the variances of burnout, depression, and anxiety, respectively.

**Conclusions:**

Work-related stressors are associated with burnout, but few are associated with anxiety and depression. Outside-of-work characteristics explained little of the distress reported by physicians. Strategies focused on perceptions of professional recognition and lower workloads that stimulate positive relationships between doctors and other health professionals are desirable in oncological context.

**Electronic supplementary material:**

The online version of this article (10.1186/s12885-018-4964-7) contains supplementary material, which is available to authorized users.

## Background

The high mortality and distress related to cancer, coupled with the increasing number of patients with cancer [1–3], places physicians who care for these patients at high risk for distress, i.e., anxiety, depression, and (in particular) burnout [4].

Burnout is a multifactorial syndrome characterized by physical and emotional exhaustion (EE), primarily catalyzed by professional demands. Burnout is characterized by high levels of EE, cynicism and depersonalization (DP; i.e., detachment or disengagement), and a decreased perception of personal fulfillment (PF) [5]. Burnout interferes with perceptions of personal well-being, increasing the risks of suicidal ideation, absenteeism, and lower medical productivity [6, 7]. Numerous studies have been published evaluating depression in medical students, interns, residents, and fellows [8–10]; however, the literature on depression and anxiety among oncologists is scarce.

Modern doctors must cope with potential stressors such as increasing government regulations of their professional activities, processes for errors/improper medical practices, briefer patient time, increased clinical demands, the commercial aspects of medicine, and the continuous expansion of scientific knowledge [11]. A high burden exists among oncologists regarding the frustration of treating patients with an incurable disease who are often distressed, in many cases angered by the situation, and experience physical-psychological-social-existential suffering. Oncologists often stand in the midst of this emotional conflict, trying to mediate between the cancer and the suffering patient.

This study sought to evaluate the prevalence of burnout, anxiety, and depression in a sample of physicians dedicated to oncology of various specialties and to identify how much of this distress could be explained by personal characteristics, those related specifically to the workplace, stressors related to work perceived by physicians, and extra-professional characteristics.

## Methods

### Place of study

The Barretos Cancer Hospital (BCH) is located in Barretos – SP (Brazil), a city with approximately 110,000 inhabitants. It is currently one of the largest cancer hospitals in Brazil, providing free care for 10,000 new cancer cases annually. BCH is an assistential, teaching, and research institution.

### Ethical aspects

The Research Ethics Committee of HCB approved the study protocol (CEP/HCB no. 1.091.484/2015). Participants indicated their agreement to participate in the study via the electronic informed consent included in the survey form.

### Study design

A cross-sectional study design with online surveys was employed.

### Casuistry

During the research period, 323 physicians (i.e., staff, residents, and fellows) worked in the hospital and were invited to participate in the study.

### Calculation of sample size

Estimates suggest that approximately 60% of the population of physicians who work in hospitals present with burnout [12, 13]. Accepting an estimate of absolute precision (i.e., how close the estimate is to the true value) of 10% and a level of significance of 1%, the minimum estimated sample size was 160 physicians [14].

#### Assessment instruments

Sociodemographic data and variables related to the work and daily lives of physicians were included in the survey.

### Maslach burnout inventory (MBI)

The MBI is composed of 22 questions answered using a seven-point Likert scale ranging from 0 (never) to 6 (every day). Of the 22 questions, nine evaluated EE, five evaluated DP, and eight evaluated PF. The classification for each dimension is given by the sum of their respective affirmations, making it possible to highlight them in low, moderate, or high levels. The PF dimension was reverse scored [15, 16]. The following cutoff values were used: EE (low level, ≤ 18; intermediate level, 19–26; high level, ≥ 27); DP (low level, ≤ 5; intermediate level, 6–9; high level, ≥ 10); and PF (low level, ≥ 40; intermediate level 39–34; high level ≤ 33). Burnout was diagnosed when high levels of EE, DP, or both were found [17]. We paid for the rights to use the MBI, and its use was duly authorized (Mind Garden, Inc., http://www.mindgarden.com/).

### Hospital anxiety and depression scale (HADS)

The HADS [18] is composed of seven items related to anxiety symptoms and seven related to the depression symptoms, totaling 14 items. All of the items are answered using a four-point Likert scale. For each dimension (anxiety/depression), cut-off scores are established for “possible cases” or “probable cases”. These scores are calculated based on the sum of the questions used to evaluate the anxiety and depression domains. In this study, scores ≥11 were considered positive for both the anxiety and depression domains.

### Questionnaire developed for the present study

The physician characteristics potentially related to burnout, anxiety, and depression were included in a questionnaire developed specifically for the present study. This questionnaire was created after meetings among the authors and a review of the literature. These characteristics were grouped into four main categories: (1) personal characteristics of physicians that are innate or difficult to modify; (2) physical or objective workplace characteristics; (3) the work-related stressors perceived by physicians; and (4) outside-of-work characteristics such as leisure, religiosity/spirituality, physical activity, and family relationships (Additional file 1**)**. Content validation was conducted with 10 physicians from different departments using cognitive debriefing and think-aloud method. All the questions were adequately understood. Two items were modified after suggestions; both suggestions were to include examples between parenthesis (regarding leisure and physical activity). An Expert Committee was formed to analyze the development of the questionnaire and the results of the pilot testing. In addition to the authors of the study, the committee was composed of one physician (MD, PhD), two experts in questionnaire validation and a psychologist. They analyzed all responses and considered the questionnaire valid to be used in the present survey. The translated English version of the questionnaire is shown in Additional file 2.

### Data collection

To apply the instruments, the web-based program *SurveyMonkey®* (https://pt.surveymonkey.com) was used. The doctors received an e-mail explaining the research and a link that provided access to the informed consent and the survey. They received three reminder e-mails about the survey each week for 3 weeks. Unanswered e-mails were considered refusal to participate.

### Statistical analyses

Variables were individually compared based on diagnoses of burnout (yes/no), depression (yes/no), and anxiety (yes/no). Categorical and continuous variables were analyzed using Fisher’s exact test and Mann-Whitney U test, respectively. Variables associated with *p* < 0.10 in the univariate analyses were included in blocks in a hierarchical binary logistic regression model to identify the predictors of burnout, depression, and anxiety. The blocks were divided a priori into (1) personal characteristics of the physicians; (2) workplace characteristics; (3) workplace-related stressors perceived by the physicians; and (4) outside-of-work characteristics. The difference in the measurement of error (− 2 log likelihood) between different blocks is the block χ^2^. Statistical tests for the estimated models (model χ^2^) and block within each model were described. In addition, we assessed the amount of variance (%) explained by the logistic models using Nagelkerke’s R^2^ parameter [19]. A two-tailed *p*-value of < 0.05 was considered significant. All statistical analyses were conducted using SPSS for Windows version 21 (SPSS, Inc., Chicago, IL, USA).

## Results

### Population description

A total of 323 physicians were invited to participate in this study; 237 accessed the research link, and four chose not to participate after reading the electronic informed consent. Of the 233 responders, the data for seven were not analyzed because those doctors did not complete at least the MBI. Thus, the final sample was composed of 227 physicians (response rate = 70.5%). A CONSORT flow diagram is described in Additional file 3.

The median age of the physicians was 34 years old (25th percentile [p25]–p75 = 30–40 years old). Most physicians (*n* = 123, 54.2%) received more than BRL 20,000 annually, were married (*n* = 140, 61.7%), and had no children (*n* = 130, 57.3%). A total of 139 (61.2%) were staff members, and 88 (38.8%) were residents or fellows. In total, 88 (38.8%) were clinicians, 63 (27.8%) were surgeons, 57 (25.1%) worked in the diagnostic sector, 11 (4.5%) were emergency or Intensive Care Unit (ICU) physicians, and eight (3.5%) were anesthesiologists **(**Additional file 4**)**.

### Burnout, depression, and anxiety scores and prevalence

**Fig. 1:**
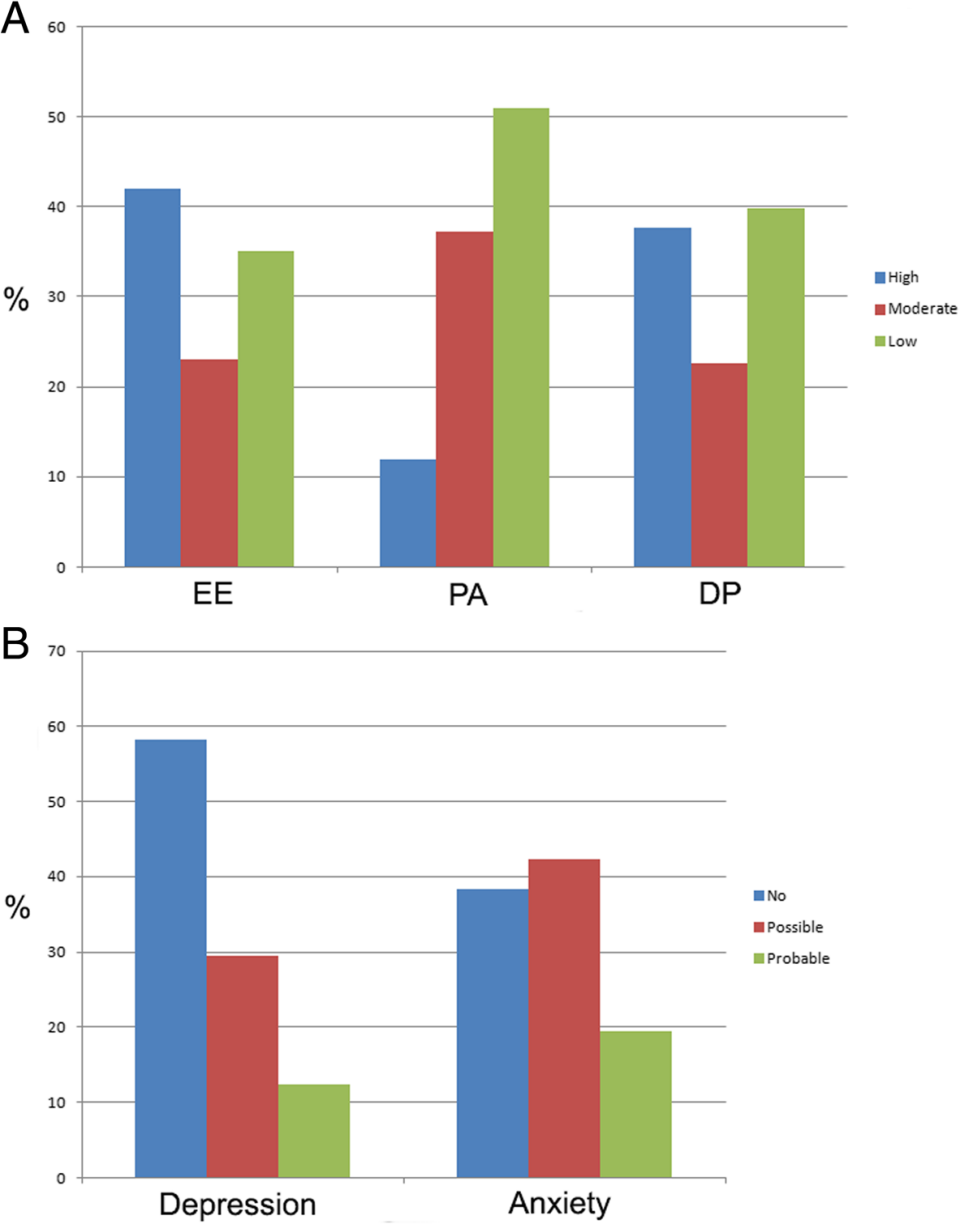
Prevalence rates (%) of burnout, depression and anxiety among physicians from a cancer hospital. **a** Burnout domains. EE: emotional exhaustion; PA: personal accomplishment; DP: depersonalization. Scores of MBI are represented in columns printed in different colors: blue, high levels; red, moderate levels; green, low levels. **b** Scores of HADS-D (depression) and HADS-A (anxiety) are divided in three categories: no (< 8, blue), possible (8–11, red), and probable (> 11, green) depression/anxiety

In total, 132 (58.1%, 95% CIs = 51.5–64.3%) physicians were identified as having burnout (i.e., EE, high DP, or both), 28 (12.3%, 95% CIs = 8.0–17.2%) had depression (HADS-D ≥ 11), and 44 (19.4%, 95% CIs = 14.6–24.8%) had anxiety (HADS-A ≥ 11). Regarding the MBI domains, 95 (41.9%, 95% CIs = 35.4–48.7%), 85 (37.6%, 95% CIs = 31.0–44.2%), and 115 (50.9%, 95% CIs = 44.2–57.1%) physicians were considered as having high EE, high DP, and low PF, respectively (Fig. 1).

The physicians were further categorized according to the median time of practice in the hospital (≤2 years vs. > 2 years). Physicians with less than 2 years of practice in the hospital presented a higher number of burnout compared to physicians with longer practice times (64.6% vs. 51.3%, *p* = 0.045). When analyzed by the burnout domain scores, an statistical significant difference was observed only in relation to EE (≤2 years, high EE = 49.1% vs. > 2 years, high EE = 34.2%, *p* = 0.012). Additional file 4: Table S2 describes the analysis of burnout in function of years of practice in the present hospital.

### Multivariate analyses

All variables with *p*-values < 0.10 in the univariate analyses (Table 1) were included in hierarchical binary logistic regression models for each analyzed outcome. The variables were included in blocks according to an a priori defined model. The final burnout regression model explained 43.6% of the total variance; blocks 1, 2, 3, and 4 individually explained 10.5%, 7.6%, 22.4%, and 3.1%, respectively. The chance of being diagnosed with burnout was higher among physicians who reported being pessimistic (OR = 7.601, *p* = 0.036), working in ICU/emergency department [ED] (OR = 6.456, *p* = 0.063), perceiving a lack of hospital recognition (OR = 2.605, *p* = 0.018), and excess work (OR = 4.735, *p* < 0.001). On the other hand, physicians who reported a higher frequency of leisure activities were less likely to be diagnosed with burnout (moderate: OR = 0.321, *p* = 0.009; frequent/very frequent: OR = 0.362, *p* = 0.065; Table 2).

**Table 1:** Variables associated with Burnout, depression and anxiety in a sample of oncologists from a large Brazilian cancer hospital

Variable	Burnout			Depression			Anxiety		
	Yes N (%)	No N (%)	*P*	Yes N (%)	No N (%)	*P*	Yes N (%)	No N (%)	*P*
Physician characteristics									
Age in years; median (P25-P75)	33 (30–39)	37 (31–42)	0.008*	34 (30–41)	32.5 (30–38.2)	0.145*	34 (30–41)	34.5 (31–39)	0.803*
Gender (male)	77 (58.3)	66 (69.5)	0.096	10 (35.7)	133 (66.8)	0.003	17 (38.6)	126 (68.9)	< 0.001
Income (Brazilian reais)			0.075			0.059			0.308
< 10.999	49 (37.1)	24 (25.3)		11 (39.3)	62 (31.2)		14 (31.8)	59 (32.2)	
11.000–19.999	20 (15.2)	11 (11.6)		7 (25.0)	24 (12.0)		9 (20.5)	22 (12.0)	
> 20.000	63 (47.7)	60 (63.2)		10 (35.7)	113 (56.8)		21 (47.7)	102 (55.7)	
Optimistic personality (no vs. yes)			0.032			0.001			0.001
Optimist	68 (51.5)	63 (66.3)		8 (28.6)	123 (61.8)		15 (34.0)	116 (63.4)	
Neither optimist nor pessimist	53 (40.2)	30 (31.6)		15 (53.6)	68 (34.2)		24 (54.5)	59 (32.2)	
Pessimist	11 (8.3)	2 (2.1)		5 (17.9)	8 (4.0)		5 (11.4)	8 (4.4)	
Psychological/psychiatric antecedents	4 (3.0)	5 (5.3)	0.497	2 (7.1)	7 (3.5)	0.307	5 (11.4)	4 (2.2)	0.015
MSc./PhD	32 (24.2)	32 (33.7)	0.136	2 (7.1)	62 (31.2)	0.007	34 (77.3)	129 (70.5)	0.457
Resident/fellow	57 (43.2)	31 (32.6)	0.129	12 (42.9)	76 (38.2)	0.681	16 (36.4)	72 (39.3)	0.863
Married	76 (57.6)	64 (67.4)	0.166	13 (46.4)	127 (63.8)	0.097	22 (50.0)	114 (62.3)	0.731
Have children	51 (38.6)	46 (48.4)	0.174	10 (35.7)	87 (43.7)	0.541	21 (47.7)	76 (41.5)	0.499
Workplace characteristics									
Main work type			0.046			0.017			0.115
Clinic	58 (43.9)	30 (31.6)		18 (64.3)	70 (35.2)		24 (54.5)	64 (35.0)	
Surgery	30 (22.7)	33 (34.7)		4 (14.3)	59 (29.6)		7 (15.9)	56 (30.6)	
Diagnosis	29 (22)	28 (29.5)		3 (10.7)	54 (27.1)		9 (20.5)	48 (26.2)	
ICU/ED	9 (6.8)	2 (2.1)		1 (3.6)	10 (5.0)		2 (4.5)	9 (4.9)	
Anesthesiology	6 (4.5)	2 (2.1)		2 (7.1)	6 (3.0)		2 (4.5)	6 (3.3)	
Years of work in the hospital (years; median, P25-p75)	2 (1–4)	3 (1–8)	0.040*	2.5 (1–7)	1.5 (0.3–3)	0.003*	2 (1–7)	2 (1–3)	0.391*
Percentual time dedicated to clinical consults			0.752			0.106			0.442
< 25%	25 (18.9)	18 (18.9)		2 (7.1)	41 (20.6)		6 (13.6)	37 (20.2)	
25–75%	36 (27.3)	30 (31.6)		12 (42.9)	54 (27.1)		16 (36.4)	50 (27.3)	
> 75%	71 (53.8)	47 (49.5)		14 (50.0)	104 (52.3)		22 (50.0)	96 (52.4)	
Working in places with higher death rates	102 (77.3)	64 (67.4)	0.129	23 (82.1)	143 (71.6)	0.362	34 (77.3)	132 (72.1)	0.573
Workplace perceived stressors									
Lack of recognition by the hospital	70 (53.0)	28 (29.5)	< 0.001	19 (67.9)	79 (39.7)	0.007	29 (65.9)	69 (37.7)	0.001
Lack of recognition by patients/caregivers	18 (13.6)	2 (2.1)	0.002	7 (25.0)	13 (6.5)	0.005	9 (20.5)	11 (6.0)	0.006
Relationship problems with other health professionals	22 (16.7)	11 (11.6)	0.342	8 (28.6)	25 (12.6)	0.040	13 (29.5)	20 (10.9)	0.004
Excess of work	78 (59.0)	19 (20.0)	< 0.001	20 (71.4)	77 (38.7)	0.002	31 (70.4)	66 (36.0)	< 0.001
Lack of time	97 (73.5)	46 (48.4)	< 0.001	24 (85.7)	119 (59.8)	0.007	35 (79.5)	108 (59.0)	0.014
Lack of resources	9 (6.8)	6 (6.3)	1.000	4 (14.3)	11 (5.5)	0.097	3 (6.8)	12 (6.6)	1.000
Institutional rules	50 (37.9)	24 (25.3)	0.062	12 (42.9)	62 (31.2)	0.281	23 (52.3)	51 (27.9)	0.004
Unawareness of the institution’s strategic plan	33 (25.0)	16 (16.8)	0.190	7 (25.0)	42 (21.1)	0.628	13 (29.5)	36 (19.7)	0.158
Lack of autonomy	27 (20.5)	7 (7.4)	0.008	6 (21.4)	28 (14.0)	0.393	10 (22.7)	24 (13.1)	0.155
Extra-work characteristics									
Frequently familiar meetings	78 (59.0)	63 (66.3)	0.332	10 (35.7)	131 (65.2)	0.003	24 (54.5)	117 (63.9)	0.299
Leisure activities			< 0.001			< 0.001			0.002
Never/rarely	58 (43.9)	17 (17.9)		19 (67.9)	56 (28.1)		24 (54.5)	51 (27.9)	
Moderate	58 (43.9)	51 (53.7)		7 (25.0)	102 (51.3)		17 (38.6)	92 (50.3)	
Frequent/very frequent	16 (12.1)	27 (28.4)		2 (7.1)	41 (20.6)		3 (6.8)	40 (21.9)	
Physical activity (no vs. yes)			0.166			< 0.001			0.022
No	46 (34.8)	22 (23.2)		17 (60.7)	51 (25.6)		21 (47.7)	47 (25.7)	
Up to 2 times a week	40 (30.3)	35 (36.8)		9 (32.1)	66 (33.2)		11 (25.0)	64 (35.0)	
More than 2 times a week	46 (34.8)	38 (40.0)		2 (7.1)	82 (41.2)		12 (27.3)	72 (39.3)	
Religion affiliation (yes)	109 (82.6)	80 (84.2)	0.857	25 (89.3)	164 (82.4)	0.588	38 (86.4)	151 (82.5)	0.656
Influence of spirituality on work (yes)	59 (44.7)	45 (47.4)	0.787	14 (50.0)	90 (45.2)	0.688	24 (54.5)	80 (43.7)	0.239

*Mann-Whitney U test

**Table 2:** Hierarchical binary logistic regression on potential variables associated with burnout in physicians from a cancer hospital

Variables	Block 1^a^		Block 2^a^		Block 3^a^		Block 4^a^	
	OR	*p*-value	OR	*p*-value	OR	*p*-value	OR	*p*-value
Block 1 - Physician characteristics								
Age; years	0.959	0.041	0.952	0.104	0.958	0.236	0.946	0.134
Optimistic personality								
Optimist	1.000		1.000		1.000		1.000	
Neither optimist nor pessimist	1.681	0.080	1.773	0.070	1.708	0.147	1.643	0.200
Pessimist	5.160	0.044	5.348	0.051	6.400	0.052	7.601	0.036
Gender (male)	0.729	0.290	0.857	0.663	1.196	0.637	1.418	0.380
Income (Brazilian reais)								
< 10.999	1.000		1.000		1.000			
11.000–19.999	1.047	0.923	1.003	0.995	0.742	0.575	0.870	0.803
> 20.000	0.877	0.737	0.711	0.417	0.399	0.063	0.492	0.168
Block 2 - Workplace characteristics								
Main work type								
Clinic			1.000		1.000		1.000	
Surgery			0.450	0.029	0.521	0.142	0.507	0.132
Diagnosis			0.392	0.016	0.627	0.288	0.660	0.364
ICU/ED			3.318	0.154	5.467	0.075	6.456	0.063
Anesthesiology			1.760	0.594	0.834	0.853	0.672	0.688
Years of work in the hospital (continuous)			0.998	0.966	1.002	0.966	1.011	0.808
Block 3 – Workplace perceived stressors								
Lack of recognition by the hospital					2.200	0.044	2.605	0.018
Lack of recognition by patients/caregivers					4.333	0.081	4.780	0.074
Excess of work					5.187	< 0.001	4.735	< 0.001
Lack of time					1.697	0.133	1.420	0.339
Institutional rules					0.967	0.932	0.824	0.627
Lack of autonomy					2.216	0.141	2.249	0.148
Block 4 - Extra-work characteristics								
Leisure activities								
Never/rarely							1.000	
Moderate							0.321	0.009
Frequent/very frequent							0.362	0.065
*Model Chi-square*	18.511	0.005	32.719	0.001	81.397	< 0.001	88.866	< 0.001
*Block Chi-square*	18.511	0.005	14.208	0.014	48.678	< 0.001	7.489	0.024
Nagelkerke’s *R*^*2*^	0.105		0.181		0.405		0.436	
*Change in* Nagelkerke’s *R*^*2*^			0.076		0.224		0.031	

^a^ In all blocks there were 132 events of burnout

The final depression regression model explained 58.4% of the total variance; blocks 1, 2, 3, and 4 individually explained 25.2%, 12.1%, 7.7%, and 13.4%, respectively. Physicians who reported being pessimistic were approximately 10 times more likely to have depression than those who reported being optimistic (OR = 10.729, *p* = 0.021). Physicians who practiced regular physical activity more than twice per week were less depressed (OR = 0.049, *p* = 0.006) than those who did not receive regular physical activity (Table 3).

**Table 3:** Hierarchical binary logistic regression on potential variables associated with depression in physicians from a cancer hospital

Variables	Block 1^a^		Block 2^a^		Block 3^a^		Block 4^a^	
	OR	*p*-value	OR	*p*-value	OR	*p*-value	OR	*p*-value
Block 1 - Physician characteristics								
Age in years	0.970	0.425	1.020	0.714	1.032	0.600	0.944	0.515
Income (Brazilian reais)								
< 10.999	1.000		1.000		1.000		1.000	
11.000–19.999	2.263	0.176	2.447	0.184	3.500	0.106	10.729	0.021
> 20.000	1.353	0.671	0.908	0.902	0.664	0.634	0.802	0.827
Gender (male vs. female)	0.271	0.005	0.328	0.025	0.302	0.034	0.284	0.055
Optimistic personality								
Optimist	1.000		1.000		1.000		1.000	
Neither optimist nor pessimist	3.679	0.008	5.087	0.002	3.187	0.045	3.034	0.104
Pessimist	10.188	0.002	14.180	0.001	13.309	0.005	18.440	0.008
MSc./PhD (yes vs. no)	0.192	0.050	0.253	0.128	0.281	0.174	0.245	0.181
Married(yes vs. no)	0.872	0.815	1.005	0.993	0.779	0.724	0.341	0.230
Block 2 - Workplace characteristics								
Main work type								
Clinic			1.000		1.000		1.000	
Surgery			0.253	0.049	0.368	0.192	0.126	0.029
Diagnosis			0.119	0.004	0.210	0.061	0.206	0.096
ICU/ED			0.371	0.411	0.247	0.329	0.500	0.698
Anesthesiology			2.182	0.476	2.372	0.463	2.014	0.593
Years of work in the hospital (continuous)			0.835	0.140	0.836	0.155	0.966	0.831
Block 3 – Workplace perceived stressors								
Lack of recognition by the hospital					2.139	0.260	2.932	0.181
Lack of recognition by patients/caregivers					1.458	0.628	1.308	0.225
Relationship problems with other health professionals					2.821	0.131	4.035	0.073
Excess of work					1.337	0.647	1.146	0.855
Lack of time					2.505	0.227	0.960	0.964
Lack of resources					2.544	0.285	7.948	0.094
Block 4 - Extra-work characteristics								
Leisure activities								
Never/rarely							1.000	
Moderate							0.254	0.097
Frequent/very frequent							0.707	0.773
Physical activity								
No							1.000	
Up to 2 times a week							0.369	0.156
More than 2 times a week							0.049	0.006
Frequently familiar meetings (yes vs. no)							0.261	0.069
*Model Chi-square*	32.286	< 0.001	49.566	0.001	61.374	< 0.001	83.322	< 0.001
*Block Chi-square*	32.286	< 0.001	17.280	0.004	11.809	0.066	21.947	0.001
Nagelkerke’s *R*^*2*^	0.252		0.373		0.450		0.584	
*Change in* Nagelkerke’s *R*^*2*^			0.121		0.077		0.134	

^a^ In all blocks there were 28 events of depression

The final anxiety regression model explained 41.2% of the total variance; blocks 1, 2, 3, and 4 individually explained 22.9%, 4.3%, 10.6%, and 3.4%, respectively. Male doctors presented with lower anxiety rates compared with female doctors (OR = 0.410, *p* = 0.052). The presence of a psychological/psychiatric history of illness (OR = 8.188, *p* = 0.017) was associated with higher anxiety rates. Regarding the work stressors perceived by physicians, relationship problems with other health professionals (OR = 3.218, *p* = 0.023) and excess work (OR = 2.396, *p* = 0.074) were associated with more anxiety (Table 4).

**Table 4:** Hierarchical binary logistic regression on potential variables associated with anxiety in physicians from a cancer hospital

Variables	Block 1^a^		Block 2^a^		Block 3^a^		Block 4^a^	
	OR	*p*-value	OR	*p*-value	OR	*p*-value	OR	*p*-value
Block 1 - Physician characteristics								
Gender (male vs. female)	0.235	< 0.001	0.261	0.001	0.326	0.012	0.410	0.052
Psychological/psychiatric antecedents	11.041	0.002	10.205	0.003	7.519	0.018	8.188	0.017
Optimistic personality								
Optimist	1.000		1.000		1.000		1.000	
Neither optimist nor pessimist	3.121	0.003	3.808	0.001	2.680	0.023	2.406	0.049
Pessimist	5.182	0.015	5.804	0.014	3.984	0.082	4.027	0.091
Block 2 - Workplace characteristics								
Main work type								
Clinic			1.000		1.000		1.000	
Surgery			0.433	0.126	0.632	0.434	0.556	0.326
Diagnosis			0.459	0.107	0.892	0.835	1.056	0.911
ICU/ED			0.518	0.472	0.525	0.520	0.530	0.533
Anesthesiology			1.116	0.907	1.417	0.719	1.223	0.838
Block 3 – Workplace perceived stressors								
Lack of recognition by the hospital					1.554	0.366	1.603	0.350
Lack of recognition by patients/caregivers					1.977	0.288	2.638	0.152
Relationship problems with other health professionals					3.144	0.023	3.218	0.023
Excess of work					2.405	0.060	2.396	0.074
Lack of time					1.510	0.417	1.237	0.686
Institutional rules					1.704	0.222	1.756	0.223
Block 4 - Extra-work characteristics								
Leisure activities								
Never/rarely							1.000	
Moderate							0.784	0.610
Frequent/very frequent							0.317	0.164
Physical activity								
No							1.000	
Up to 2 times a week							0.400	0.093
More than 2 times a week							0.594	0.311
*Model Chi-square*	35.093	< 0.001	42.442	< 0.001	61.332	< 0.001	67.654	< 0.001
*Block Chi-square*	35.093	< 0.001	7.349	0.196	18.890	0.004	6.321	0.176
Nagelkerke’s *R*^*2*^	0.229		0.272		0.378		0.412	
*Change in* Nagelkerke’s *R*^*2*^			0.043		0.106		0.034	

Controlled for age and years of practice in the hospital

^a^ In all blocks there were 44 events of anxiety

## Discussion

This study evaluated the prevalence of the distress conditions burnout, depression, and anxiety using evaluation instruments and cutoff points that have been widely used in previous studies. Approximately 58%, 12%, and 19% of physicians who treat patients with cancer show burnout, depression, and anxiety, respectively. In addition, we identified distress predictors among oncologists. Clearly, a considerable proportion of oncologists should be cared for in addition to caring for their patients. To the best of our knowledge, this is the first study to use hierarchical regression models in order to evaluate factors associated with distress conditions in oncologists. Of the factors evaluated, issues related to the perception of stressors at work explained burnout best; in turn, these same stressors had little importance with regard to anxiety and depression.

The mental health of physicians is a relevant topic. Previous studies conducted with physicians from distinct countries showed rates of depressive symptoms varying from 8.8 and 28.1% [20–25]. In a Chinese study [25], a sample of 2641 physicians showed a 25.6% prevalence of anxiety symptoms. The rates of anxiety and depression in the present study are compatible with those in the literature; however, if we had used cutoff points for mild symptoms, then we would have found rates greater than those in the literature (i.e., ranging from 30 to 40%).

At least 50% of North American physicians have burnout [26, 27]. Among the various medical specialties, those who treat patients entering the health system (e.g., general practitioners and internists) have a higher incidence of burnout [26]. A recent meta-analysis showed that 32% of oncologists have high levels of burnout [4]. In our study, almost 60% of physicians were identified as having burnout; those who worked in intensive care or emergency medicine were most affected. We believe that working in sectors with potentially serious cases increases the risk of burnout. However, working in places with higher death rates was not associated with a higher prevalence of burnout. Thus, it is possible that caring for patients with cancer who have an indication for invasive measures, but not necessarily those with advanced cancer in palliative care (without an indication of invasive measures), predicts the development of burnout. The rates of depression and anxiety in our study are consistent with a recent meta-analysis, which showed that 27% of oncologists have psychiatric comorbidities, and at least 12% test positive for depression.

Burnout is recognized as a work-related problem, and the organizational environment plays a critical role in its development [28]. Physicians’ perceptions of their supervisors’ leadership qualities are correlated with burnout and job satisfaction [29]. However, the association between anxiety, depression, and occupational aspects is less obvious. In fact, we observed that physicians’ perceptions of their stressors at work explained only about 5% and 10% of the total variance of depression and anxiety, respectively. Promoting advancement in one’s professional career, guidance, and recognition of the results obtained are some of the strategies employed to reduce burnout rates among physicians [30]. Caruso et al. identified lack of recognition as a significant organizational stressors in a cancer hospital in Italy [31]. Similar results were obtained among medical ophthalmologists [32] and anesthesiologists [33]. Workload, time pressure, pressure for efficiency, role conflicts, lack of control over work, lack of support from supervisors and co-workers, little participation in decision making, lack of autonomy, and challenges with the work-life balance are common work-related factors associated with burnout [16, 28].

This study has limitations. The first is that it is a cross-sectional study, and it is impossible to determine cause-and-effect relationships. The second is that we evaluated work stressors based on the opinions of physicians and did not objectively measure their number of appointments or actual working time. However, we believe that perceptions of one’s work, and not necessarily the work itself, are most important with regard to the genesis of burnout. Another limitation is that we did not evaluate the number of deaths that each physician witnessed in his or her daily life; rather, we arbitrarily divided the workplaces into those with greater or fewer deaths.

Screening for emotional distress among physicians who treat patients with cancer is currently mandatory [28]. In particular, burnout cannot be considered a problem only for the doctor; rather, it is a shared responsibility with the hospital. Individual-focused treatment strategies (e.g., stress management and self-care training, communication skills training, and mindfulness-based approaches) or workplace or organizational changes (e.g., briefer attending rotation lengths, various modifications to clinical work processes, and practice delivery changes) are effective. However, one must define the most effective strategies for specific populations [34]. Healthcare managers must recognize that the well-being of their workers is an essential goal, as are the satisfaction and improvement of the health of their patients, without disregarding the costs involved. Physicians must be mentally prepared for proper engagement and production.

## Conclusions

In summary, approximately 12%, 22%, and 60% of oncologists experience depression, anxiety, and burnout. Work-related stressors are associated with burnout, but few are associated with anxiety or depression. Outside-of-work characteristics explain little of the distress reported by physicians. Strategies focused on the perception of professional recognition and lowered workloads (which stimulate positive relationships between doctors and other health professionals) are desirable.

## Additional files

Additional file 1: **Figure S1.** Conceptual model representing possible physician’s distress-related categories. A: physician characteristics; B: Workplace characteristics; C: Workplace perceived stressors; D: Extra-work characteristics. (TIF 15367 kb)

Additional file 2: Questionnaire used in the present study. (DOC 44 kb)

Additional file 3: **Figure S2.** CONSORT flow diagram. (TIF 24 kb)

Additional file 4: **Table S1.** Demographic characteristics of the physicians. **Table S2.** Analysis of burnout in function of years of practice in the present hospital. (DOC 70 kb)

## Abbreviations

BCH: Barretos Cancer Hospital
DP: depersonalization
ED: emergency department
EE: emotional exhaustion
HADS: Hospital Anxiety and Depression Scale
ICF: informed consent form
ICU: Intensive Care Unit
MBI: Maslach Burnout Inventory
PF: personal fulfillment
SD: standard deviation
