# Supplementary figures and images for: Doctor, are you healthy? A cross-sectional investigation of oncologist burnout, depression, and anxiety and an investigation of their associated factors
Source: BMC Cancer. 2018 Oct 26;18:1044. doi: 10.1186/s12885-018-4964-7 (PMC6203972; doi:10.1186/s12885-018-4964-7)

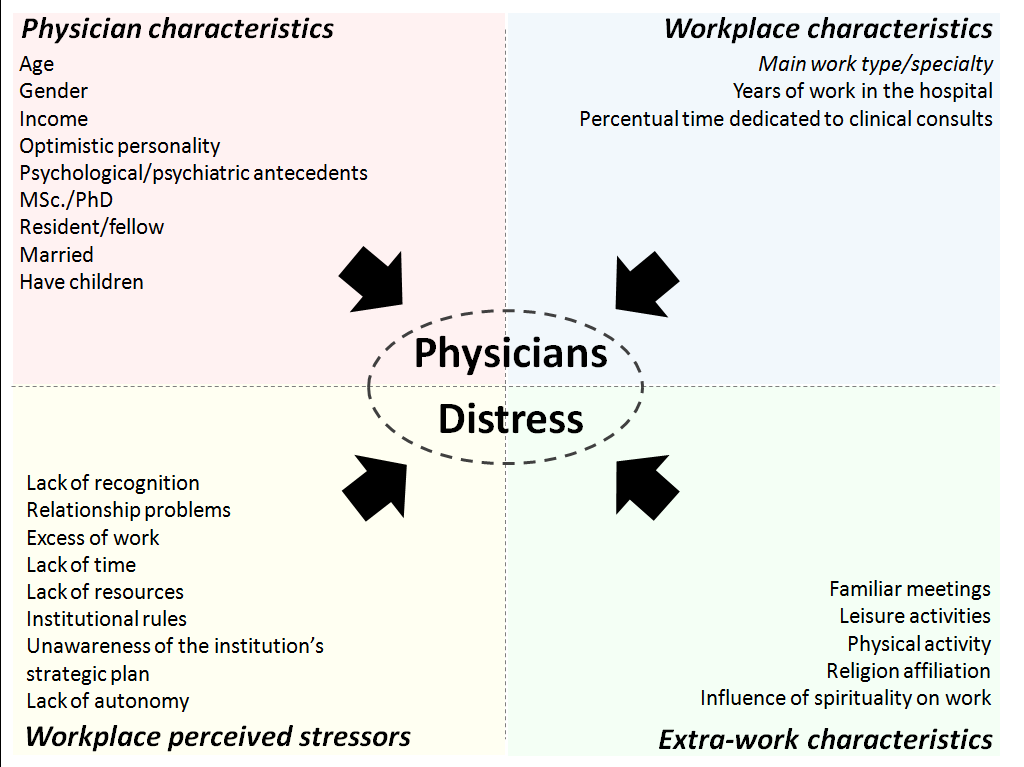

Supplement: Supplementary file 1 — Figure S1. Conceptual model representing possible physician’s distress-related categories. A: physician characteristics; B: Workplace characteristics; C: Workplace perceived stressors; D: Extra-work characteristics. (TIF 15367 kb) [file 12885_2018_4964_MOESM1_ESM.tif]

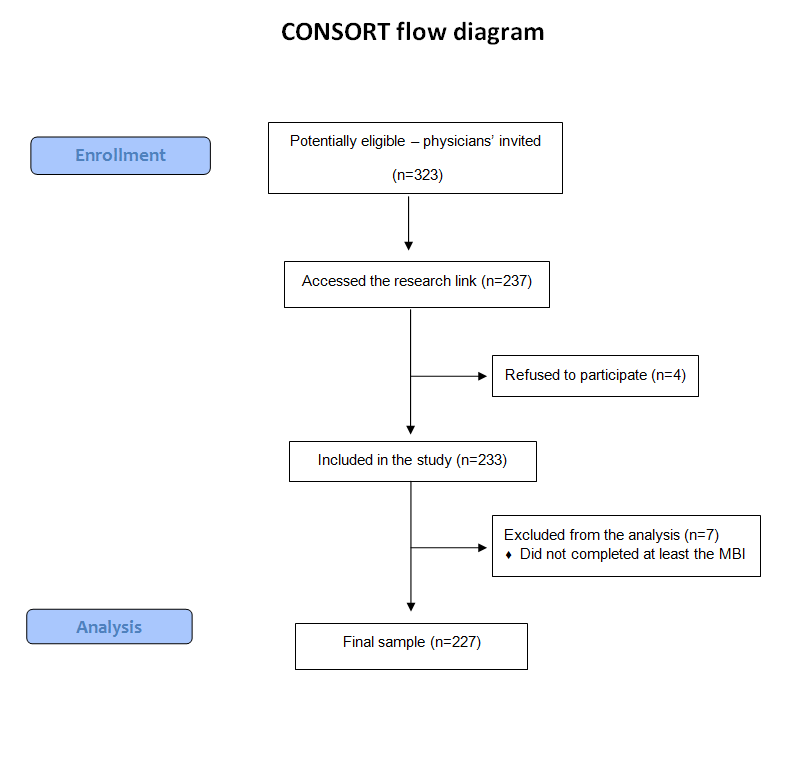

Supplement: Supplementary file 3 — Figure S2. CONSORT flow diagram. (TIF 24 kb) [file 12885_2018_4964_MOESM3_ESM.tif]
