# Supplementary material for: Doctor, are you healthy? A cross-sectional investigation of oncologist burnout, depression, and anxiety and an investigation of their associated factors
Source: BMC Cancer. 2018 Oct 26;18:1044. doi: 10.1186/s12885-018-4964-7 (PMC6203972; doi:10.1186/s12885-018-4964-7)
Supplement: Supplementary file 2 — Questionnaire used in the present study. (DOC 44 kb) [file 12885_2018_4964_MOESM2_ESM.doc]

**Additional file 2**

**Physician characteristics**

**1. Check below what is your gender:**

( ) female ( ) male

**2. How old are you?____________ years**

**3. You consider yourself...**

( ) Optimistic

( ) Neither optimistic nor pessimistic

( ) Pessimistic

**4. Do you have any psychiatric or psychological health problem?**

( ) yes

( ) no

**5. What is your educational level? (You can check more than one option)**

( ) medical residence

( ) Specialization (Lato Sensu)

( ) Master, PhD or post-doc

( ) Other (please specify)

**6. What is your current marital status?**

( ) Married or living as married

( ) widowed

( ) divorced

( ) single

**7. Do you have kids?**

( ) yes

( ) no

**8. How many kids do you have?**

( ) 1

( ) 2

( ) 3

( ) 4

( ) 5

( ) 6

**Workplace characteristics**

**9. What department or area do you currently work in most of the time?**

( ) Clinical oncology

( ) Breast surgery

( ) Digestive surgery (upper)

( ) Digestive surgery (low)

( ) Radiology

( ) Pathology

( ) Emergency

( ) Endoscopy

( ) Intensive care

( ) Palliative care

( ) Thoracic surgery

( ) Pediatrics

( ) Head and neck surgery

( ) Prevention

( ) Urology

( ) Skin and soft tissue surgery

( ) Infectious diseases

( ) Gynecology surgery

( ) Other (please specify)

**10. How many years have you been working as a doctor at Barretos Cancer Hospital?**

_____________________ years

**11. From your time at the Barretos Cancer Hospital, what percentage do you spend for direct patient care (office visits, clinical procedures, etc)?**

( ) 0%; because I do not provide direct care to patients

( ) <25% of working time

( ) 25% - 75% of working time

( ) >75% of working time

**Workplace perceived stressors**

**12. From the following situations, which has caused you professional discomfort or dissatisfaction? (you can mark more than one)**

( ) Lack of recognition by the hospital

( ) Lack of recognition by patients/caregivers

( ) Relationship problems with members of the multiprofessional team

( ) Excess of work

( ) Lack of time to carry out other activities at work (research, technical improvements, schedules for study, etc)

( ) Lack of resources for proper treatment of patients

( ) Institutional rules

( ) Unawareness of the institution’s strategic plan

( ) Lack of autonomy

**Outside-of-work characteristics**

**13. How often do you and your family meet at home for a “family meeting” (for example, lunch and family dinner)?**

( ) never

( ) rarely

( ) sometimes

( ) often

( ) Always

**14. To what extent have you had leisure time (going out for walks, moments of rest, talking, traveling, theater, cinema, etc.)?**

( ) Not at all

( ) Slighly

( ) More or less

( ) Very much

( ) Extremely

**15. How often have you been performing some kind of physical activity (walking, cycling, soccer, swimming, some kind of fighting, etc.)?**

( ) I do not do physical activity

( ) 1 time per week

( ) 2 times per week

( ) 3 times per week

( ) 4 times per week

( ) 5 times per week

( ) 6 times per week

( ) 7 times per week

**16. Do you have a religion?**

( ) I do not have

( ) Catholic

( ) Evangelical

( ) Spiritist

( ) Other (please specify): ________________________________

**17. To what extent does your religious or spiritual life have influence in your professional life?**

( ) Not at all

( ) Slighly

( ) More or less

( ) Very much

( ) Extremely

*This questionnaire was translated literally from Portuguese into English, thus it cannot be considered ready to be used in English language.*
