# Supplementary material for: Doctor, are you healthy? A cross-sectional investigation of oncologist burnout, depression, and anxiety and an investigation of their associated factors
Source: BMC Cancer. 2018 Oct 26;18:1044. doi: 10.1186/s12885-018-4964-7 (PMC6203972; doi:10.1186/s12885-018-4964-7)
Supplement: Supplementary file 4 — Table S1. Demographic characteristics of the physicians. Table S2. Analysis of burnout in function of years of practice in the present hospital. (DOC 70 kb) [file 12885_2018_4964_MOESM4_ESM.doc]

**Additional File 4**

**Table S1.** Demographic characteristics of the physicians (n=227)

| **Variable** | **N** | **%** |
| --- | --- | --- |
| ***Physician characteristics*** |  |  |
| Age in years; median ( P25-P75) | 34 (30-40) | |
| Gender (male) |  |  |
| Male | 143 | 63.0 |
| Female | 84 | 37.0 |
| Income (reais) |  |  |
| <10.999 | 73 | 32.2 |
| 11.000 – 19.999 | 31 | 13.7 |
| >20.000 | 123 | 54.2 |
| Civil status |  |  |
| Single | 81 | 35.7 |
| Married | 140 | 61.7 |
| Separated | 6 | 2.6 |
| Have children |  |  |
| No | 130 | 57.3 |
| 1 | 34 | 15.0 |
| 2 or more | 53 | 27.8 |
| Physician’s role |  |  |
| Staff member | 139 | 61.2 |
| Resident/fellow | 88 | 38.8 |
| MSc or PhD degrees |  |  |
| No | 163 | 71.8 |
| Yes | 64 | 28.2 |
| ***Workplace characteristics*** |  |  |
| *Main work type* |  |  |
| Clinic¹ | 88 | 38.8 |
| Surgery | 63 | 27.8 |
| Diagnosis² | 57 | 25.1 |
| ICU/ED | 11 | 4.8 |
| Anesthesiology | 8 | 3.5 |

¹ Clinical oncology, radiation therapy, Palliative care, cardiology, oncogenetics. ² Radiololgy, Pathology, Nuclear Medicine, Endoscopy.

**Table S2.** Analysis of burnout in function of years of practice in the present hospital.

| **Burnout** | **Category levels** | **Years of practice in the hospital** | | **p-value** |
| --- | --- | --- | --- | --- |
|  |  | **≤2 years**  **n (%)** | **>2 years**  **n (%)** |  |
| Emotional exhaustion |  |  |  | 0.012 |
|  | Low | 30 (25.9) | 49 (44.1) |  |
|  | Intermediate | 29 (25.0) | 24 (21.6) |  |
|  | High | 57 (49.1) | 38 (34.2) |  |
| Depersonalization |  |  |  | 0.070 |
|  | Low | 38 (32.8) | 52 (47.3) |  |
|  | Intermediate | 31 (26.7) | 20 (18.2) |  |
|  | High | 47 (40.5) | 38 (34.5) |  |
| Personal fulfillment |  |  |  | 0.109 |
|  | Low | 67 (57.8) | 49 (44.1) |  |
|  | Intermediate | 36 (31.0) | 48 (43.2) |  |
|  | High | 13 (11.2) | 14 (12.6) |  |
| Burnout |  |  |  | 0.045 |
|  | Yes | 75 (64.7) | 57 (51.4) |  |
|  | No | 41 (35.3) | 54 (48.6) |  |
